# Serum Interleukin-18 at Commencement of Renal Replacement Therapy Predicts Short-Term Prognosis in Critically Ill Patients with Acute Kidney Injury

**DOI:** 10.1371/journal.pone.0066028

**Published:** 2013-05-31

**Authors:** Chan-Yu Lin, Chih-Hsiang Chang, Pei-Chun Fan, Ya-Chung Tian, Ming-Yang Chang, Chang-Chyi Jenq, Cheng-Chieh Hung, Ji-Tseng Fang, Chih-Wei Yang, Yung-Chang Chen

**Affiliations:** Kidney Research Center, Department of Nephrology, Chang Gung Memorial Hospital, Chang Gung University College of Medicine, Taipei, Taiwan; Glaxo Smith Kline, Denmark

## Abstract

**Background:**

Acute kidney injury (AKI) requiring renal replacement therapy (RRT) in critically ill patients results in a high hospital mortality. Outcome prediction in this selected high-risk collective is challenging due to the lack of appropriate biomarkers. The aim of this study was to identify outcome-specific biomarkers in this patient population.

**Methodology/Principal Findings:**

Serum samples were collected from 101 critically ill patients with AKI at the initiation of RRT in intensive care units (ICUs) of a tertiary care university hospital between August 2008 and March 2011. Measurements of serum levels of cystatin C (CysC), neutrophil gelatinase-associated lipocalin, and interleukin-18 (IL-18) were performed. The primary outcome measure was hospital mortality. The observed overall mortality rate was 56.4% (57/101). Multiple logistic regression analysis indicated that the serum IL-18 and CysC concentrations and Acute Physiology and Chronic Health Evaluation III (ACPACHE III) scores determined on the first day of RRT were independent predictors of hospital mortality. The APACHE III score had the best discriminatory power (0.872±0.041, *p*<0.001), whereas serum IL-18 had the best Youden index (0.65) and the highest correctness of prediction (83%). Cumulative survival rates at 6-month follow-up following hospital discharge differed significantly (*p*<0.001) for serum IL-18 <1786 pg/ml vs. ≥1786 pg/ml in these critically ill patients.

**Conclusions:**

In this study, we confirmed the grave prognosis for critically ill patients at the commencement of RRT and found a strong correlation between serum IL-18 and the hospital mortality of ICU patients with dialysis-dependent AKI. In addition, we demonstrated that the APACHE III score has the best discriminative power for predicting hospital mortality in these critically ill patients.

## Introduction

Acute kidney injury (AKI) is well recognized for its impact on the outcome of patients admitted to intensive care units (ICUs). When AKI requiring renal replacement therapy (RRT) occurs, hospital mortality is high, exceeding 50% [1]–[4]. Mortality rates have changed little over the past few decades despite the significant advances in dialytic and intensive care technology [5]. This could be due to the increasing age of the patients, higher comorbidities, and/or greater illness severity. Outcome prediction in this selected high-risk collective is challenging due to the lack of appropriate biomarkers and the limited value of severity-of-illness scoring systems. Thus, the identification of outcome-specific biomarkers in this patient population is a major goal in critical care nephrology [6].

Recent studies in the field of early detection of AKI have proposed many biomarkers for the early detection of AKI. A systematic review of publications, which evaluated the accuracy and reliability of serum and urinary biomarkers in human subjects when used to diagnose the established AKI or early AKI, or to risk stratify patients with AKI, indicated that serum cystatin C (CysC), urine interleukin-18 (IL-18), and urine kidney injury molecule-1 (KIM-1) performed best for the differential diagnosis of established AKI. Serum CysC and urine neutrophil gelatinase-associated lipocalin (NGAL), IL-18, glutathione-S-transferase-π, and γ-glutathione-S-transferase performed best for the early diagnosis of AKI. Urine *N*-acetyl-β-d-glucosaminidase, KIM-1, and IL-18 performed best for mortality risk prediction after AKI [7].

Although these biomarkers have a defined role in the early detection of AKI, little is known about the diagnostic and prognostic utility of these biomarkers in patients with established AKI requiring RRT. Therefore, we aimed to prospectively evaluate the correlation between different biomarkers (serum CysC, NGAL, and IL-18) and scoring systems [Sequential Organ Failure Assessment (SOFA), Acute Physiology and Chronic Health Evaluation II (APACHE II) and APACHE III scores] at the commencement of RRT in critically ill patients and subsequent outcome.

## Materials and Methods

### Ethics statement

The study protocol was approved by the institutional review board of Chang Gung Memorial Hospital. Written informed consent was obtained from the patients or their next of kin in this study.

### Patient information and data collection

This is a prospective study, conducted between August 2008 and March 2011 in ICUs at a tertiary care referral center in Taiwan. The critically ill patients with AKI requiring RRT were enrolled. Exclusion criteria were as follows: patients or their next of kin who were unable or declined to provide written informed consent; pediatric patients (ages ≤18 years); patients with any dialysis treatment before admission to the ICU; patients with end-stage renal failure; duration of ICU stay after first RRT *<*24 h; and patients who had undergone renal transplantation. Readmitted patients were also excluded from this study.

The RRT modality was chosen by combining the clinical judgment of the consulting nephrologist with the input of the attending critical care physician. Patients who were perceived to be hemodynamically stable were treated with IHD. Hemodynamically unstable patients were typically prescribed CRRT. Serum samples for quantification of CysC, NGAL, and IL-18 were available from all patients at the start of the RRT. Prospectively collected data were as follows: demographics; reason for ICU admission and RRT; primary diagnosis; routine chemistry tests; SOFA and APACHE II and III scores on the first day of RRT in the ICU; duration of hospitalization; and outcome. The primary study outcome was hospital mortality. Follow-up at 6 months after hospital discharge was performed via chart record or a telephone interview.

### Sampling and quantification of serum CysC, NGAL and IL-18

Blood samples were collected in nonheparinized tubes immediately before initiation of RRT and were centrifuged at 1500 rpm for 5 minutes. Serum samples were subsequently stored at −80°C until assayed. Serum CysC and NGAL were measured in duplicate by single ELISA (R&D Systems, Minneapolis, MN, USA). Serum IL-18 was measured in duplicate by single ELISA (Medical and Biologic Laboratories, Nagoya, Japan), according to the manufacturer's instructions.

### Definitions

Severe sepsis was defined according to modified American College of Chest Physicians and Society of Critical Care Medicine consensus criteria [8]. Patients with proven or suspected infection, two or more systemic inflammatory response syndrome criteria and an infection-induced organ dysfunction were classified as having severe sepsis. Septic shock was diagnosed when the systolic arterial blood pressure remained less than 90 mmHg despite adequate fluid resuscitation. Patients with AKI on the first day of RRT were classified as Risk of renal failure, Injury to the kidney, Failure of kidney function, Loss of kidney function, and End-stage renal failure (RIFLE) according to risk, injury, and failure categories, respectively [9]. Illness severity was assessed by using the APACHE II and III scores [10], [11] and the SOFA score [12]. The worst physiological and biochemical values on the day of initial RRT in the ICU were recorded. These systems have been validated to predict the outcomes of critically ill patients [13], [14]. The treatment of RRT in critically ill patients was indicated on the basis of clinical grounds, including refractory hyperkalaemia, resistant fluid overload, severe persistent metabolic acidosis, and overt uremic symptoms, including uremic pericarditis and encephalopathy [15].

### Statistical analysis

Descriptive statistics are expressed as means ± standard error (SE). Primary analysis compared hospital survivors with non-survivors. All variables were tested for normal distribution using the Kolmogorov-Smirnov test. Student's *t-*test was applied to compare the means of continuous variables and normal distribution data. Otherwise, the Mann-Whitney *U* test was employed. Categorical data were tested using the Chi-square test. We assessed the risk factors for hospital mortality by using univariate analysis, and the variables that were found to be statistically significant (*p*<0.05) in the univariate analysis were included in the multivariate analysis. Multiple logistic regression model based on the forward elimination of data was used to analyze these variables.

Hosmer-Lemeshow goodness-of-fit test was used for calibration when evaluating the number of observed and predicted deaths in risk groups for the entire range of death probabilities. Discrimination was assessed using the area under the receiver operating characteristic curve (AUROC), which was compared using a nonparametric approach. The AUROC analysis was also performed to calculate cutoff values, sensitivity, and specificity. Finally, cutoff points were calculated by acquiring the best Youden index [16]. The index is defined as sensitivity + specificity −1, where sensitivity and specificity are calculated as proportions. Youden index has minimum and maximum values of −1 and +1, respectively, with a value of +1 representing the optimal value for an algorithm.

Cumulative survival curves as a function of time were generated using the Kaplan-Meier approach and compared using the log-rank test. All statistical tests were two-tailed; a value of *p<*0.05 was considered statistically significant. Data were analyzed using SPSS (SPSS, Inc., Chicago, IL, USA).

## Results

### Subject characteristics

Between August 2008 and March 2011, 101 patients with AKI requiring RRT in ICUs were enrolled in this study. The 101 consecutive critically ill patients with AKI were treated with continuous renal replacement therapy (CRRT) (n = 27) or intermittent hemodialysis (IHD) (n = 74). No patients met the RIFLE criteria for loss or end-stage renal disease categories. Patient median age was 60 years; 68 were male (67%) and 33 were female (33%). Overall, hospital mortality for the entire group was 56.4% (57/101). Of the 57 non-survival patients, 52 patients (91.2%) had sepsis and 19 patients (33.3%) had liver cirrhosis (Table 1). Table 2 describes the reasons for ICU admission and indications for RRT. The most frequent indications for RRT were anuria/oliguria.

**Table 1:** Comparison of hospital survivors and non- survivals after commencement of renal replacement therapy.

	All Patients (n = 101)	Survivors (n = 44)	Non-survivors (n = 57)	*p*-value
Age (years)	60±2	60±3	58±2	NS (0.736)
Length of hospital stay (day)	47±4	57±6	39±5	0.031
Male, *n* (%)	68 (67)	29 (66)	39 (68)	NS (0.790)
RRT modality (IHD), *n* (%)	74 (73)	36 (82)	38 (67)	NS (0.088)
Diabetes mellitus, *n* (%)	30 (30)	16 (36)	14 (25)	NS (0.198)
Hypertension, *n* (%)	29 (29)	16 (36)	13 (23)	NS (0.135)
CKD, *n* (%)	43 (43)	22 (50)	21 (37)	NS (0.185)
Sepsis, *n* (%)	73 (72)	21 (48)	52 (91)	<0.001
Liver cirrhosis, *n* (%)	20 (20)	1 (2)	19 (33)	<0.001
Body weight (kg)	63±1	63±2	64±2	NS (0.893)
Serum Cys C (mg/L)	4.9±0.2	4.0±0.3	5.7±0.3	<0.001
Serum IL-18 (pg/mL)	2179±149	1265±79	2873±211	<0.001
Serum NGAL (ng/mL)	795±66	513±42	1012±104	<0.001
PaO_2_/FiO_2_	218±10	228±16	212±14	NS (0.464)
GCS (points)	12.6±0.4	13.3±0.5	12.2±0.5	NS (0.122)
MAP (mmHg)	84±2	88±3	82±3	NS (0.168)
Serum Creatinine (mg/dL)	4.8±0.2	4.7±0.3	4.9±0.3	NS (0.207)
Urine output (mL/day)	343±44	369±41	327±68	NS (0.652)
Sodium (mEq/L)	139±1	139±1	139±1	NS (0.980)
Leukocytes (x10^3^/µL)	16.6±1.1	15.5±1.2	17.3±1.6	NS (0.438)
Hemoglobin (g/dL)	9.6±0.2	9.8±0.4	9.4±0.2	NS (0.494)
CRP (mg/L)	123±10	123±16	124±20	NS (0.972)
RIFLE (Risk/Injury/Failure)	5/15/81	3/9/32	2/6/49	NS (0.124)
SOFA (mean ± SE)	11.1±0.4	9.2±0.4	12.4±0.5	<0.001
APACHE II (mean ± SE)	18.8±0.7	16.8±1.1	20.1±0.9	0.026
APACHE III (mean ± SE)	93.2±2.2	77.2±1.4	103.3±2.7	<0.001

APACHE, Acute Physiology and Chronic Health Evaluation; CKD, chronic kidney disease; CRP, C reactive protein; CysC, cystatin C; FiO_2_, fraction of inspired oxygen; GCS, Glasgow coma scale; IHD, intermittent hemodialysis; IL-18, interleukin-18; MAP, mean arterial pressure; NGAL: neutrophil gelatinase-associated lipocalin; NS, not significant; PaO2, partial pressure of oxygen; RIFLE, Risk of renal failure, Injury to the kidney, Failure of kidney function, Loss of kidney function, and End-stage renal failure; RRT, renal replacement therapy; SE, standard error; SOFA, sequential organ failure assessment.

**Table 2:** Primary diagnosis for intensive care unit admission and renal replacement therapy indication between hospital survivors and non-survivors.

	All Patients (n = 101) n (%)	Survivors (n = 44) n (%)	Non-survivors (n = 57) n (%)	*p*
*Primary ICU admission*				
Respiratory failure with ventilator	39 (39)	20 (45)	19 (33)	NS (0.215)
Septic shock	8 (8)	0 (0)	8 (14)	0.010
Severe UGI bleeding	11 (11)	5 (11)	6 (11)	NS (0.894)
Acute decompensated liver failure	14 (14)	1 (2)	13 (23)	0.003
Postoperative care	18 (18)	12 (27)	6 (11)	0.029
Others a	11 (11)	6 (14)	5 (9)	NS (0.437)
*Primary reason for RRT*				
Anuria/Oliguria (>12 hours)	61 (60)	24 (55)	37 (65)	NS (0.291)
AKI in progression	22 (22)	10 (23)	12 (21)	NS (0.840)
Oliguria/AKI with metabolic acidosis	7 (7)	1 (2)	6 (11)	NS (0.105)
Oliguria/AKI with hyperkalemia	5 (5)	4 (9)	1 (2)	NS (0.164)
Oliguria/AKI with pulmonary edema	6 (6)	5 (11)	1 (2)	NS (0.083)

AKI: acute kidney injury; ICU: intensive care unit; RRT, renal replacement therapy; UGI, upper gastrointestinal.

a Multifactor related.

### Hospital mortality and short-term prognosis

Table 3 lists goodness-of-fit, as measured by the Hosmer-Lemeshow chi-square statistic of predicted hospital mortality risk, the predictive accuracy of serum CysC, IL-18, and NGAL as well as APACHE II and III and SOFA scores. We also compared the discriminatory value of these AKI biomarkers and the three scoring systems. The AUROC analysis verified that the APACHE III score had the best discriminatory power.

**Table 3:** Comparison of calibration and discrimination of the biomarkers and scoring systems on the first day of renal replacement therapy in predicting hospital mortality.

	Calibration			Discrimination		
	Hosmer-Lemeshow χ^2^	df	*p*	AUROC ± SE	95% CI	*p*
Serum CysC	15.458	8	0.051	0.771±0.055	0.662–0.880	<0.001
Serum IL-18	7.865	8	0.447	0.849±0.039	0.772–0.926	<0.001
Serum NGAL	7.872	8	0.446	0.765±0.055	0.658–0.931	<0.001
SOFA	11.750	8	0.163	0.767±0.054	0.661–0.872	<0.001
APACHE II	8.803	8	0.359	0.644±0.063^a^	0.521–0.767	0.024
APACHE III	12.389	8	0.134	0.872±0.041	0.792–0.951	<0.001

APACHE, Acute Physiology and Chronic Health Evaluation; AUROC, areas under the receiver operating characteristic curve; CI, confidence intervals; CysC, cystatin C; df, degree of freedom; IL-18, interleukin-18; NGAL: neutrophil gelatinase-associated lipocalin; RRT, renal replacement therapy; SE, standard error; SOFA, sequential organ failure assessment.

a , *p*<0.05 vs. Serum IL-18 and APACHE III.

**Figure 1:**
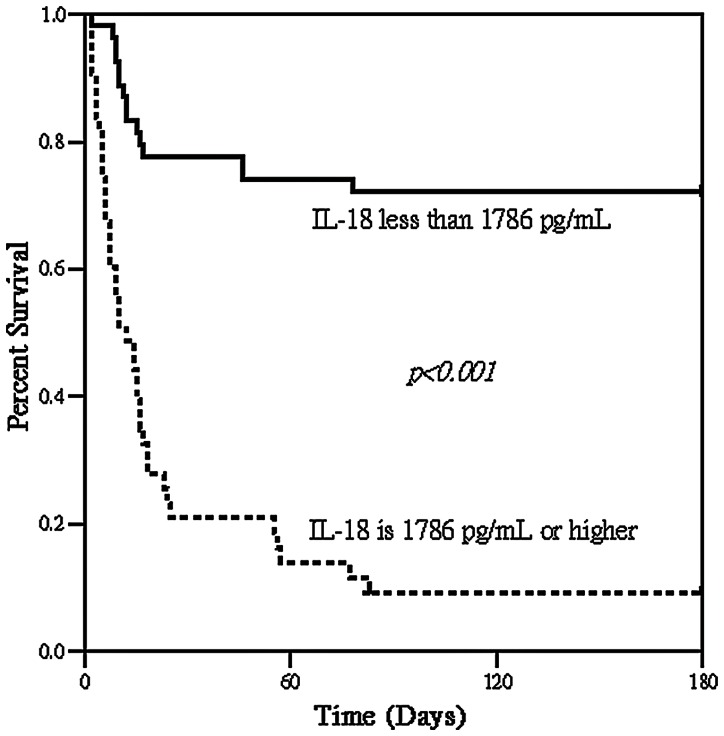
Cumulative survival for 101 critically ill patients with acute kidney injury requiring renal replacement therapy (RRT) according to a below and above cut-off of serum interleukin-18 (IL-18) level 1786 pg/mL measured before the initiation of RRT.

To assess the predictive value of selected cut-offs for predicting hospital mortality, the sensitivity, specificity, and overall correctness of prediction were determined. Table 4 summarizes the data calculated using the cutoff point providing the best Youden index. Serum IL-18 had the best Youden index and the highest overall correctness of prediction. The overall 1-month, 3-month and 6-month mortality rates were 48.5% (49/101), 57.4% (58/101) and 57.4% (58/101) respectively. 1-month, 3-month and 6-month mortality rates significantly differed below and above the cutoff point of serum IL-18  = 1786 pg/mL (21.8% *vs.* 80.4%, *p*<0.001; 27.3% *vs.* 93.5%, *p*<0.001 and 27.3% *vs.* 93.5%, *p*<0.001 respectively). Figure 1 illustrates that 6-month cumulative survival rates differed significantly (*p*<0.001) between the two groups divided by the cutoff point of serum IL-18  = 1786 pg/mL in the study population.

**Table 4:** Subsequent hospital mortality predicted on the commencement of renal replacement therapy.

Predictive Factors	Cutoff Point	Youden Index	Sensitivity (%)	Specificity (%)	Overall Correctness (%)
Serum CysC (mg/L)	4.3^a^	0.49	79	70	75
Serum IL-18 (pg/mL)	1786^a^	0.65	77	88	83
Serum NGAL (ng/mL)	873^a^	0.42	51	91	71
SOFA	13^a^	0.46	57	89	73
APACHE II	15^a^	0.22	67	55	61
APACHE III	96^a^	0.60	71	89	80

APACHE, Acute Physiology and Chronic Health Evaluation; CysC, cystatin C; IL-18, interleukin-18; NGAL: neutrophil gelatinase-associated lipocalin; RRT, renal replacement therapy; SOFA, sequential organ failure assessment.

a Value giving the best Youden index.

Univariate analysis identified 8 (Table 5) out of 25 variables (Table 1, except the length of hospital stay) as prognostically valuable. Multivariate analysis identified the following variables as having independent prognostic significance: serum IL-18 and CysC levels and APACHE III score (Table 5). Regression coefficients of these variables were utilized to calculate a logit of death for each patient as follows:

**Table 5:** Logistic regression analysis for hospital mortality, according to baseline prognostic factors on the first day of renal replacement therapy.

Parameter	Beta coefficient	Standard error	Odds ratio (95%CI)	*p*
*Univariate logistic regression*				
Sepsis	2.501	0.559	12.190 (4.076–36.452)	<0.001
Liver cirrhosis	3.019	1.050	20.462 (2.614–160.151)	0.004
Serum CysC	0.480	0.134	1.615 (1.242–2.101)	<0.001
Serum IL-18	0.002	0.000	1.002 (1.001–1.003)	<0.001
Serum NGAL	0.002	0.001	1.002 (1.001–1.004)	0.001
SOFA	0.253	0.075	1.288 (1.112–1.492)	0.001
APACHE II	0.081	0.036	1.084 (1.011–1.163)	0.024
APACHE III	0.116	0.025	1.123 (1.069–1.180)	<0.001
*Multivariate logistic regression*				
Serum CysC	0.802	0.282	2.229 (1.283–3.872)	0.004
Serum IL-18	0.001	0.001	1.001 (1.000–1.002)	0.008
APACHE III	0.142	0.057	1.152 (1.031–1.288)	0.012
Constant	−18.052	5.662	-	-

APACHE, Acute Physiology and Chronic Health Evaluation; CI, confidence intervals; CysC, cystatin C; IL-18, interleukin-18; NGAL: neutrophil gelatinase-associated lipocalin; RRT, renal replacement therapy; SOFA, sequential organ failure assessment.

The logarithm of death odds  = −18.052+0.802× serum CysC +0.001× serum IL-18 +0.142× APACHE III.

## Discussion

Several studies have identified a mortality rate of more than 50% in critically ill patients with AKI requiring RRT [1]–[4]. The hospital mortality rate for patients in the present study was 56.4%. The analytical results verify that prognosis is grave for this patient subgroup. This study also confirmed that APACHE III score and serum IL-18 and CysC levels on RRT day 1 were strongly correlated with hospital mortality.

A large study of healthy, middle-aged European men showed that serum IL-18 concentration is an independent predictor of coronary events [17]. In addition, variation within the IL-18 gene is known to influence the circulating concentrations of IL-18 and clinical outcome in patients with coronary heart disease [18]. IL-18 is an important regulator of both innate and acquired immune responses [19]. It is present in human atherosclerotic lesions, and at higher concentrations in unstable plaques [20]. In animal models, IL-18 administration leads to increases in atherosclerotic lesion size and promotes increased numbers of T-lymphocytes in the lesion [21], suggesting that it may be of pathogenic importance in tissues. The authors of this study group previously demonstrated that APACHE II score, serum sodium concentrations, urinary NGAL, and serum IL-18 on day 1 of coronary care unit admission are independent predictors of 180-day mortality [22]. In the present study, serum IL-18 level showed significantly better discriminatory power than the APACHE II score at the initiation of RRT for hospital mortality prediction (Table 3). Serum IL-18 also had the best Youden index and the highest overall correctness of prediction (Table 4). Furthermore, patients with serum concentrations of IL-18 ≥1786 pg/mL on RRT day 1 were associated with an extremely high half-year mortality rate of 93% (40/43) (Figure 1).

Cystatin C is a 13-kDa protein that is normally freely filtered, completely reabsorbed, and catabolized within the proximal tubule [23]. It is a marker of glomerular filtration and has performed extremely well in identifying established AKI. In patients with AKI, serum cystatin C level performs similarly to serum creatinine level, serum urea nitrogen level, and urine output in predicting dialysis requirement or in-hospital death [24]. Urinary cystatin C was independently associated with death within 30 days in critically ill patients [25]. However, the predictive utility of CysC in critically ill patients with established AKI requiring RRT is poorly characterized. Apart from the serum concentration of IL-18, this study has demonstrated that APACHE III score and serum CysC level are independently associated with hospital mortality.

Because AKI occurs in patients with varying profiles and diverse disease etiologies, predicting the outcome of patients with AKI requiring RRT is difficult. The APACHE system assumes that the core mission of intensive care is treating disease and maintaining physiological homeostasis. Physiological abnormalities are common among ICU patients, and the extent of derangement is an objective and reproducible measure of illness severity [26]. In this study, the APACHE III score, but not the APACHE II score, showed good calibration and the best discriminatory power, thus indicating the superior prognostic accuracy of APACHE III when compared with the other scoring systems evaluated in this patient population. Moreover, the pathogenesis of experimental AKI secondary to endotoxemia is the compensation for impaired hemodynamics by up-regulation of vasoconstrictor systems and renal vasoconstriction [13], [27]. Although septic AKI is an independent predictor of mortality, the leading causes of death associated with AKI are non-renal complications, typically those related to multi-organ dysfunction.

Despite the promising results obtained in this study, several important limitations should be recognized. First, this study was conducted in a single institution. Consequently, the results may not be directly extrapolated to other patient populations. Second, predictions vary among individuals. Accordingly, a prediction is only an approximate indicator of mortality risk in specific subjects. Third, we examined scoring systems only during the first day of RRT support, although these models were developed and calculated on the first day of ICU admission. Fourth, liver cirrhosis was largely attributed to hepatitis B viral infection (55%) in this investigation. The number of patients with cirrhosis (n = 20) and outcome events were insufficient to determine independent risk factors for hospital mortality by using multivariate techniques. Finally, sequential measurement of these AKI biomarkers or other AKI biomarkers (e.g., kidney injury molecule-1, liver fatty acid-binding proteins) and scoring systems (*e.g.,* daily, weekly) may reflect the dynamic aspects of clinical diseases and thus provide superior information on mortality risk.

This study has confirmed the grave prognosis for critically ill patients with AKI requiring RRT. It has also elucidated that the predictors of APACHE III score, and serum CysC and IL-18 concentrations on the first day of RRT are independently associated with hospital mortality. Because of the relatively small sample size used in this study, the predictive roles of APACHE III score and serum CysC and IL-18 levels require further external validation.
